# Integrated hepatology and addiction care for inpatients with alcohol use disorder improves outcomes: a prospective study

**DOI:** 10.1097/HC9.0000000000000119

**Published:** 2023-04-26

**Authors:** Rachael Mahle, Paige McLean Diaz, Chantelle Marshall, Russell P. Goodman, Esperance Schaefer, Jay Luther

**Affiliations:** 1 MGH Alcohol Liver Center, Massachusetts General Hospital, Harvard Medical School, Boston, Massachusetts; 2 Division of Gastroenterology, Massachusetts General Hospital, Harvard Medical School, Boston, Massachusetts

## Abstract

Growing literature highlights the need to integrate hepatology and addiction care to improve outcomes for patients with alcohol use disorder and alcohol-associated liver disease. However, prospective data for this approach are lacking.

**Methods:**

We prospectively examined the efficacy of an integrated hepatology and addiction medicine approach on alcohol use and hepatology outcomes in inpatients with alcohol use disorder.

**Findings:**

An integrated approach improved the uptake of medical alcohol therapy, hepatic fibrosis screening, and viral hepatitis vaccination compared with a historical control of patients who received addiction medicine care alone. There were no differences in the rates of early alcohol remission. The integration of hepatology and addiction care may improve outcomes in patients with alcohol use disorder.

## INTRODUCTION

A major obstacle in the treatment of patients with alcohol-associated liver disease (ALD) is early diagnosis, as many patients with ALD are diagnosed late in their disease course.^1^ In addition, many patients at high risk of developing ALD are not on alcohol use disorder (AUD) pharmacotherapy.^2^ To address these gaps, our group developed a dedicated inpatient alcohol liver evaluation (ALivE) team to evaluate patients with AUD for subclinical ALD while admitted to the hospital for non–liver-related complaints. This consultation service was paired with the robust inpatient addiction consultation team, which has previously been reported to improve outcomes in AUD.^3^ The previous descriptive analysis of the ALivE service demonstrated its efficacy for identifying ALD in admitted patients and promoting engagement in outpatient care.^4^ However, prospective data evaluating the efficacy of a collaborative hepatology and addiction medicine service are missing.

**FIGURE 1:**
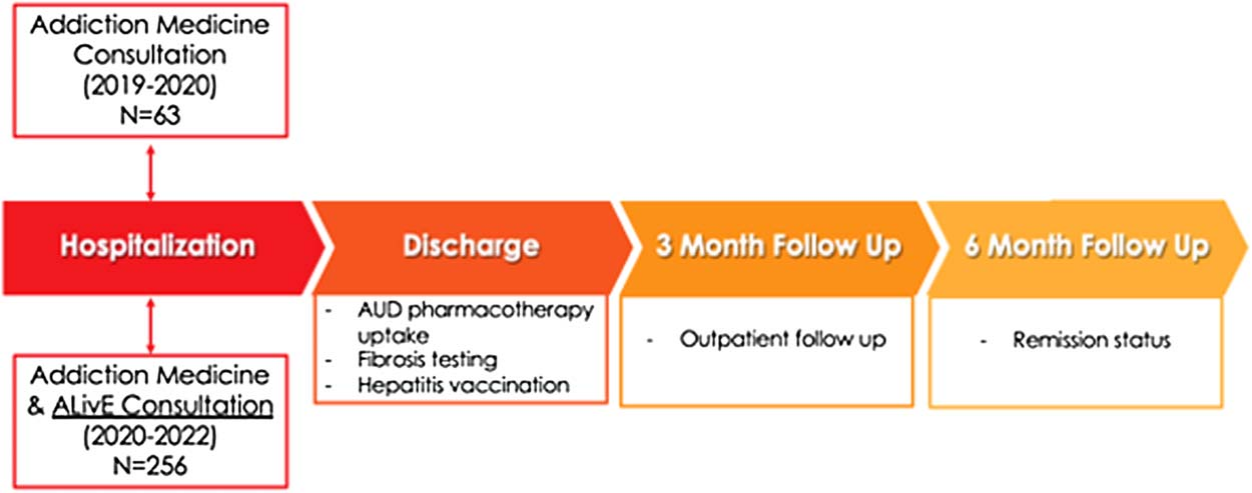
Schematic of the study design. Patients with AUD without known liver disease admitted to the hospital for non–liver-related complaints were enrolled during hospitalization. The treatment group was evaluated by both addiction medicine and ALivE. The standard-of-care group consisted of a historical cohort of patients with AUD admitted 1 year before the creation of ALivE and therefore were evaluated by addiction medicine alone. Important liver-related outcomes at the time of discharge and 3- and 6-month follow-up were analyzed. Abbreviations: AUD, alcohol use disorder; ALiVE, alcohol liver evaluation team.

This single-center, prospective study evaluated the performance of a novel multidisciplinary consultation group (ALivE plus addiction medicine) against a historical cohort of patients with AUD who received standard of care (SOC) in our hospital system. Starting in January 2020, primary inpatient medical services were provided the option to consult the ALivE service (which consisted of 1 hepatologist and a nurse practitioner) for patients with AUD admitted to the hospital with no known liver disease or evidence of current liver disease. These patients were evaluated by the ALivE service and underwent fibrosis staging with elastography, discussion of the importance of viral hepatitis vaccination, and education of the deleterious effects of alcohol on liver health. All patients seen by the ALivE team were also evaluated by addiction medicine.^4^ The SOC control group consisted of patients with AUD, and no known previous or active liver disease (based on chart documentation, laboratory, and imaging analysis) admitted to the hospital from January 2019 to January 2020, 1 year before the ALIVE consultation started. These patients received a standard of care at that time, which included management by the addiction medicine consultation service alone. We approached all identified patients with AUD admitted from January 2019 to January 2020 for inclusion into the SOC group; however, only those patients who consented to enter our alcohol biorepository patient cohort were included in the SOC group so that we could follow their clinical course without actively being involved in their care. Demographic and clinical data were extracted from patients’ electronic medical records. AUD pharmacotherapy included any 1 of 3 medications approved by the US Food and Drug Administration for AUD (naltrexone oral or intramuscular, disulfiram, and acamprosate), as well as medications used off-label for AUD (gabapentin, topiramate, or baclofen). Patients who had one of these medications prescribed during their hospitalization and/or on their hospital discharge summary were counted as treated. Early remission was defined by alcohol abstinence at a 6-month follow-up from study enrollment (Figure 1), per patient report documented in the electronic medical records and not based on objective laboratory data. Hepatitis A and B vaccination administration was assessed through chart review. Subjects without any health care encounters after the time of enrollment, missing data, or those lost to follow-up were excluded from the remission analysis. Continuous variables were summarized with means (SDs) and compared using an unpaired, 2-tailed *t* test with Welch correction, whereas categorical variables were compared using the Fisher exact test.

In total, 256 patients were included in the ALivE group, whereas 63 patients were included in the SOC group (Table 1). Patients evaluated by the ALivE service were more likely to be Hispanic and less likely to speak English, be married, or have a concurrent substance use diagnosis compared with the SOC group. The most common reason for hospital presentation in both cohorts was alcohol intoxication or withdrawal. Patients evaluated by ALivE had higher rates of liver fibrosis screening compared with SOC (84.8% vs. 1.6%; *p* < 0.001), leading to the identification of F2 or greater fibrosis by noninvasive testing in 25.8% (N = 66) of the ALivE cohort. We found that patients in the ALivE cohort had higher rates of AUD pharmacotherapy prescriptions at discharge compared with the SOC group (73.4% vs. 57.1%; *p* = 0.012). The rate of new AUD therapy (newly prescribed during the hospitalization or at discharge) was higher in the ALivE group compared with the SOC group (67.3% vs. 37.8%, *p* < 0.001). With regard to remission data, 189 of the 256 patients seen by the ALiVE service had data available at the 6-month follow-up time point compared with 45 of the 63 patients in the SOC group. We did not see a difference in either early remission (13.9% vs. 14.3%, *p* = 0.819) or partial remission (less than a 6-month period of abstinence in the 6-month follow-up period; 51.3% vs. 44.4%, *p* = 0.407) between the ALiVE and SOC groups, respectively. As expected, given the involvement of a hepatologist in the patient’s care, the ALivE cohort had higher rates of hepatitis A (49.6% vs. 11.1%; *p* = 0.04) and hepatitis B (51.0% vs. 17.6%; *p* < 0.01) vaccination in nonimmune patients.

**TABLE 1:** Characteristics of included patients

	Intervention group (ALivE)	Standard of care	*p*
Sex, N (%)			
Male	197 (77)	49 (77.8)	0.89
Female	59 (23)	14 (22.2)	0.81
Age, years (SD)	49.5 (12.3)	55.3 (13.1)	<0.01
Ethnicity, N (%)			
Hispanic	51 (19.9)	4 (6.3)	0.01
Non-Hispanic	196 (76.6)	54 (85.7)	0.12
Marital status, N (%)			
Married/partnered	57 (22.3)	22 (35.0)	0.03
Divorced/separated	80 (31.3)	13 (20.6)	0.11
Single	119 (46.5)	27 (42.9)	0.68
Admitting diagnosis, N (%)			
Alcohol withdrawal/alcohol intoxication	125 (48.8)	15 (23.8)	<0.01
Pancreatitis	15 (1.6)	5 (7.9)	0.54
Infection (not SBP)	10 (5.9)	4 (6.3)	0.40
GI complaint (nausea, constipation, diarrhea)	17 (6.6)	4 (6.3)	0.93
GI bleed	8 (3.1)	2 (3.2)	0.98
Cardiovascular/dyspnea	18 (7.0)	15 (23.8)	<0.01
Neurologic/psychiatric	17 (6.6)	5 (7.9)	0.72
Metabolic abnormality (including ketosis)	10 (3.9)	1 (1.6)	0.66
Fall/injury	13 (5.1)	9 (14.3)	0.01
Anemia	4 (1.6)	0	—
COVID-19	6 (2.3)	0	—
Other	13 (5.1)	3 (4.8)	0.64
BMI±SD (kg/m^2^)	28.1±5.9	26.5±5.3	0.44
AST/ALT±SD (units/L)	2.0**±**1.2	1.7**±**0.8	<0.01
Alkaline phosphatase±SD (IU/L)	121.8±69.8	92.6±31.7	<0.01
Total bilirubin±SD (µmol/L)	0.98±0.6	0.62±0.4	<0.01
Substance use history (other than alcohol), N (%)			
No history of substance use	192 (75.0)	27 (45.8)	<0.01
History of substance use	64 (25.0)	32 (54.2)	<0.01
No current substance use	224 (87.5)	39 (63.9)	<0.01
Current substance use	32 (12.5)	22 (36.1)	<0.01
Positive hepatitis C antibody, N (%)	39 (15.2)	8 (12.6)	0.61
Public insurance, N (%)	119 (46.5)	18 (28.6)	0.01
English as primary language, N (%)	163 (63.7)	62 (98.4)	<0.01

Abbreviations: ALivE, alcohol liver evaluation; ALT, alanine transaminase; AST, aspartate aminotransferase; BMI, body mass index; GI, gastrointestinal; SBP, spontaneous bacterial peritonitis.

In this prospective cohort study, we found that the evaluation of patients with AUD without known liver disease by a hepatologist improved diagnostic and therapeutic care compared with the SOC. We found that hepatology evaluation increased liver fibrosis screening and advanced fibrosis identification, improved rates of AUD pharmacotherapy prescription, and improved rates of preventative hepatology care. The SOC at our hospital for patients with any substance use disorder is the evaluation by the addiction service, which provides education, behavior coaching, and substance use treatments. The findings of our study suggest that an integrated model of addiction medicine and hepatology could further improve care for patients with AUD in a multimodal capacity.^5^ However, certain limitations must be highlighted. For one, the ALiVE group differed in multiple ways from the SOC group, which may affect our observed results. Second, we did have a considerable dropout rate during our 6-month follow-up period. Finally, during the time-period when ALiVE consultation was available (January 2020–2022), there were many patients who qualified for ALiVE consult that did not receive one.^4^ This could be related to patient or provider preference. Nonetheless, it could bias our results to favor the involvement of patients more willing to seek care.

Despite the evaluation by addiction medicine, an integrated consult approach with hepatology resulted in higher rates of AUD pharmacotherapy at the time of discharge. This finding is particularly important, given that data show an association between AUD pharmacotherapy and reduced odds of developing ALD in those without advanced fibrosis or having a decompensating hepatic event in those with underlying cirrhosis. The underutilization of AUD pharmacotherapy is well recognized,^6^ and our results highlight the impact that hepatology evaluation and counseling may have on the acceptance of AUD treatment.
